# Phenotype, Sex of Rearing, Gender Re-Assignment, and Response to Medical Treatment in Extended Family Members with a Novel Mutation in the SRD5A2 Gene

**DOI:** 10.4274/jcrpe.2782

**Published:** 2016-06-06

**Authors:** Asma Deeb, Hana Al Suwaidi, Fakunle Ibukunoluwa, Salima Attia

**Affiliations:** 1 Mafraq Hospital, Clinic of Pediatric Endocrinology, Abu Dhabi, United Arab Emirates; 2 Gulf University Faculty of Medicine, Ajman, United Arab Emirates

**Keywords:** 5-alpha reductase-2, reductase deficiency, ambiguous genitalia, gender re-assignment

## Abstract

Deficiency of steroid 5-alpha reductase-2 (5ARD2) is an inborn error of metabolism causing a disorder of sexual differentiation. It is caused by a mutation in the SRD5A2 gene in which various mutation types have been reported. Affected individuals have a broad spectrum of presentation ranging from normal female-appearing genitalia, cliteromegaly, microphallus, hypospadias, to completely male-appearing genitalia. We report an extended Emirati family with 11 affected members. The family displayed various phenotypes on presentation leading to different sex of rearing. Some family members were reassigned gender at various stages of life. The index case was born with severe undervirilization with bilaterally palpable gonads and was raised as male from birth. He had a 46,XY karyotype and a high testosterone/dihydrotestosterone ratio. Genetic investigation revealed a novel homozygous deletion of exon 2 of the SRD5A2 gene. Both parents were found to be carriers for the gene deletion. The patient had masculinizing surgery and a course of topical dihydrotestosterone. No beneficial effect of the hormone application was noted over 3 months and the treatment was discontinued. The findings on this kindred indicate that deletion of exon 2 in the SRD5A2 gene causes various degrees of genital ambiguity leading to different sex of rearing in affected family members. Gender reassignment may be done at various ages even in conservative communities like the Gulf region.

## WHAT IS ALREADY KNOWN ON THIS TOPIC?

5-alpha reductase deficiency is a known cause of male undervirilization. Virilization at puberty is a common presentation and might cause patients to re-assign to male gender if they were raised as females at birth. Over 61 mutations have been reported in the SRD5A2 gene.

## WHAT THIS STUDY ADDS?

The study is the first extended family research to be reported from the Gulf region with genetically-confirmed diagnosis of 5-alpha reductase deficiency. A novel mutation is described in the reported family. Male gender re-assignment is not uncommon even in conservative communities like Arabs in the Gulf area.

## INTRODUCTION

Deficiency of steroid 5-alpha reductase-2 (5ARD2) is an inborn error of metabolism inherited in an autosomal recessive pattern. The enzyme defect results in a disorder of sexual differentiation (DSD) wherein patients with 46,XY genotype have impaired virilization during embryogenesis (1). The defect results in impaired conversion of testosterone to dihydrotestosterone (DHT). Affected individuals have a broad spectrum of presentation which may include normal female-appearing genitalia, cliteromegaly, microphallus, hypospadias, or completely male-appearing genitalia (2). This syndrome was first described clinically and biochemically in 1974 in studies of 24 affected subjects from 13 families in a large Dominican kindred (3). To date, more than 61 mutations, in the gene which codes for this isoenzyme of 5-alpha reductase called SRD5A2, have been identified to be on chromosome 2p23 (4).

There is uncertainty with regard to sex of rearing in children born with genital ambiguity in this disorder. Similar to other disorders of sexual differentiation, multiple factors beside the external genital phenotype come to play (5). Sex reassignment has been widely reported in this condition and many others. Money et al (6) reported better adjustment to sex reassignment if it was done prior to 27 months of age. Eleven of 14 children adjusted to the change without complications when the reassignment occurred prior to age 27 months, in contrast to only 1 of 4 children who adjusted to the change without complications if it occurred after 27 months.

Genital surgery is widely performed for children with genital ambiguity. Penile construction remains a challenging task for surgeons. However, some newer techniques offer improvement in males with severe micropenis and aphalia (7). Medical treatment has been tried on this enzyme defect by using topical DHT with variable rates of success (8,9). Encouraging results with topical DHT in preparation for genital surgery have been reported (10).

In this paper, we report a large Emirati kindred with high consanguinity rate and many affected members. We highlight the variable phenotypes that led to different gender assignment and re-assignment. We also report our experience in using topical DHT in the severe form of undervirilization.

## CASE REPORT

**Figure 1:**
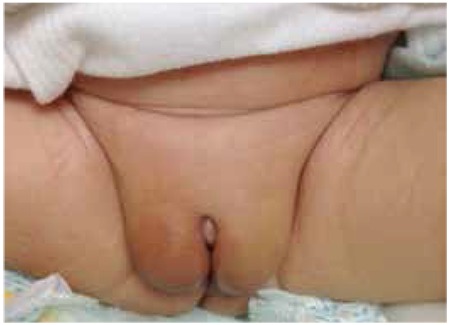
Genitalia of index case at birth (twin 2)

Our index case was a male infant, a baby born as the 2nd twin at 37 weeks with an elective cesarean section. His birth weight was 2.6 kg. It was the mother’s first pregnancy during which she had not received any medications. A postnatal examination revealed that the patient had an apparently normal external female genitalia but was also noted to have bilaterally palpable gonads in the labial folds (Figure 1). Phallus/clitoral length was around 0.5 cm. Twin 1 had normal male external genitalia with bilateral palpable gonads in the scrotum. The index case is an Emirati baby who was born to a first degree cousin Emirati parents.

Ultrasound scan of the indexd case showed absent mullerian duct structures, adrenal glands of normal size, and presence of gonads in the labial folds. Initial investigations showed normal adrenal androgen levels, and normal levels for random cortisol, adrenocorticotropic hormone, and gonadotropin. Basal serum testosterone was 11.4 nmol/L [normal range (NR): 0.5-3.0] while DHT was 0.34 nmol/L. Testosterone/ DHT ratio was 33.6. Fluorescence in situ hybridization analysis on metaphases and interphase of 300 cells showed a normal male pattern of hybridization. Karyotype was 46,XY with positive SRY gene marker. Genetic studies showed a homozygous deletion of exon2 of SRD5A2 gene. Both parents were carriers. The parents decided to raise the baby as a male from birth.

**Figure 2:**
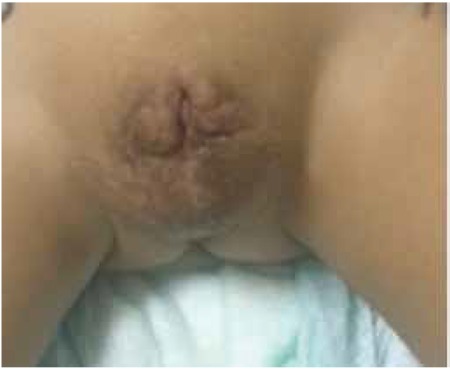
Genitalia of index case at 18 months after surgery and treatment with dihydrotestosterone cream

The patient had genital surgery at the age of 6 months when he had construction of the scrotal sac. By the age of 14 months, he received a course of percutaneous 2.5% DHT once nightly for 3 months. No response in terms of improving phallus size was noted. The child developed pubic hair at the age of 16 months and the parents stopped the medication (consent was obtained from parents to show the genital pictures) (Figure 2). Although the parents were instructed on the proper way of applying the DHT, appearance of pubic hair on the scrotal sac might indicate that the application was mainly over the genital skin rather than the phallus. Nonetheless, as the phallus was extremely small, it was believed that lack of response was possibly due to the severity of the defect rather than the imperfect application. The parents confirmed their compliance to the treatment and following the exact instruction given by the physicians on the use of DHT.

**Figure 3:**
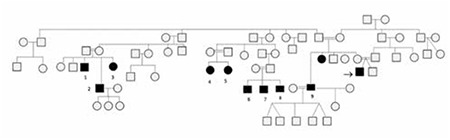
Affected family members are indicated with black squares or circles. All affected members are 46,XY. Squares indicate current male gender assignment, while circles indicate female assignment

In addition to our index patient, 7 affected family members were born with varying degree of genital ambiguity (Table 1, Figure 3). In addition to the index case, 3 members (cases 4, 5, and 9) presented with apparently normal female genitalia with palpable gonads at labial folds. All patients (except for patients 7 and 8) were raised as females. 5 patients had virilization at puberty (patients 1, 2, 3, 9, 10). Three of them reassigned their gender as males (1,2,9), while 2 (patients 3, 10) kept the female gender. Patient 10 expressed a male gender identity but was satisfied with her gender role as female in the community. Patients 4 and 5 were raised as females and are currently pre-pubertal. Parents of patients 6, 7, 8 witnessed the abnormalities in the older extended relatives and were aware of the gender re-assignment at puberty in some family members. They raised patients 6 and 7 as males and re-assigned patient 8 into a male sex at the age of 2. Patient 2 fathered 3 healthy girls after a non-consanguineous marriage. Patients 9 married twice and fathered 5 healthy boys from one marriage and a healthy girl from another.

## DISCUSSION

5-alpha reductase is an enzyme which exhibits 2 isoforms: types 1 and 2, out of which the type 2 coded by SRD5A2 on 2p23 is the predominant isoenzyme which is required for full masculinization of the fetal external genitalia (11). Type 1, which is coded by SRD5A1 gene on 5p15, expressed from the time of puberty, is responsible for virilization in type-2 isoenzyme deficient individuals (12). The 5ARD2 enzyme is responsible for the conversion of testosterone to DHT. In the fetus, both testosterone and DHT bind to the same androgen receptor protein inside the nucleus of the cell. However, they exert different physiological stimuli; testosterone has a major role in stimulation of the Wolffian ducts during sexual differentiation and control over spermatogenesis, while DHT is required for the development of normal male external genitalia (12). It is also to be noted that DHT is considered as the essential androgen as it also facilitates most of the changes of male puberty including facial, body, and genital hair appearance and prostate growth (12).

SRD5A2 deficiency is often suspected in infants with ambiguous genitalia or when adolescents, who have previously ascribed the female gender, present with marked masculinization and/or phallic growth at puberty (13). The clinical features of this disease are highly variable owing to different mutations within the same gene. It is also known for patients with the same mutations to show a wide spectrum of phenotypes (14). Often the external genitalia are female at birth. However, external genitalia may also present as complete male with microphallus and varying degrees of hypospadias, female genitalia with clitoromegaly, or normal female genitalia. The position of the testes also varies although most of the time they are found outside of the abdominal cavity in the inguinal canals or the labia majora or the scrotum (2). Our index case presented with severe undervirilization and was thought to be a female newborn at birth due to the complete appearance of female genitalia until the postnatal examination when gonads were palpated in the labial folds. The other 10 affected family members had a different phenotype of genital abnormalities. Four of them presented with apparently normal female external genitalia with palpable gonads in the labial fold and others had a varying degree of genital ambiguity.

Biochemical analysis in infants with ambiguous genitalia usually reveals a normal serum testosterone level with an elevated serum T:DHT (testosterone:dihydrotestosterone) ratio of more than 20 (2). The cut-off for this ratio is debatable for different age groups. Walter et al (11) recommend a cut-off value of 8.5 for a stimulated T:DHT estimation in young infants to avoid a false exclusion. Urinalysis may also reveal excretion patterns of 5-alpha to 5-beta reduced steroids. Occasionally, the disease can be confirmed by detecting a mutation in the SRD5A2 gene in the presence of a normal T:DHT ratio (14). In our patient, the T:DHT ratio was very high at 33. It was considered sufficient for the biochemical diagnosis and human chorionic gonadotropin test was not required to test the stimulated levels of the different androgens. Subsequently, the diagnosis was confirmed by detecting the SRD5A2 gene mutation.

SRD5A2 deficiency is more prevalent than expected in the adult female 46,XY DSD population (15). It is not uncommon for XY individuals with 5-alpha reductase deficiency reared as female to reverse gender assignment at puberty (16,17). Imperato-McGinley et al (18) interviewed affected 46,XY subjects and reported that 17 of 18 subjects with this disorder had, successfully, changed gender identity from female to male. In our kindred, 3 patients were reassigned to male sex at puberty. On interviewing a 4th individual at 42 years of age, she declared her tendency to a male gender identity and role but was not able to reverse gender as she found herself unable to acknowledge maleness. Also, she felt quite satisfied with her role as a successful female manager in the community. As to patients 6, 7, and 8, their parents decided on male sex of rearing for 2 of their affected children at birth and they re-assigned the 3rd child to male sex at the age of 2. Their decision was based on their experience on progress of other affected members in the extended family.

Male gender reassignment has been encouraged in this disorder due to the normal psychosexual development and normal genital virilization in those who converted to the male sex. In addition, fertility has been reported to be normal in this group of patients (10). In our kindred, 2 men fathered children. Patient number 9 married twice and had 6 healthy children. The other patient (number 2) also fathered 3 healthy children.

The SRD5A2 gene has over 61 known mutations reported to date. Inherited in an autosomal recessive fashion, many affected individuals are homozygotes associated with high degree of consanguinity, however, this disorder is known to exist in compound heterozygotes as well (19). Some mutations are more common in certain ethnic groups, which could be due to a founder effect (20). The SRD5A2 gene consists of 5 exons separated by 4 introns (12). Mutations of all the exons have been reported so far (14). Exons 1 and 4 were the most frequently encountered sites for mutation in a cohort of 55 patients (12). In our family, the SRD5A2 sequence analysis revealed a homozygous deletion of exon 2 in the index case. Both parents were carriers of the mutation. As of now, this mutation has not been reported. Various deletions have been reported in the 5SRD2 gene among the three largest kindred with 5ARD2 deficiency in the world: the Dominican, New Guinean, and Turkish kindred. In 2 related patients diagnosed with SRD5A2 deficiency in the Highlands of Papua New Guinea, deletion of most of the SRD5A2 gene was detected (21). In addition, Boudon et al (22) reported a trinucleotide deletion straddling codons 156 and 157, responsible for a methionine residue deletion at position 157 of the protein in a Turkish patient. Mutations in exon 2 have been reported. An adenine (GAC) for guanine (GGC) change in exon 2 causing a substitution of aspartic acid for glycine at amino acid 115 (G115D) was detected in 1 family (19).

Topical DHT treatment has been tried in various forms of undervirilization with variable degree of success (8,9). Our index case has also received topical treatment for a few weeks, but the parents stopped it due to lack of phallus growth and appearance of pubic hair. Destruction of the phallus tissue due to surgery was also thought of as a possible reason for non-responsiveness to the DHT. However, the main surgical work was done for construction of the scrotal sac and correction of the hypospadias. There was no attempts for phalloplasty at this stage. Moreover, as the family was highly consanguineous, the digenic inheritance was considered as another possible reason for the poor response to DHT (23) particularly in combination with a defect like androgen insensitivity syndrome. However, as the detected mutation clearly explained the various phenotype seen in the family members, no further genetic analysis was sought.

In conclusion, we believe that this is the first report on an extended family with 5-alpha reductase deficiency caused by a novel deletion of exon 2 of the SRD5A2. Our kindred displayed a variable genital appearance at birth among affected members. Male sex re-assignment was chosen in half of the members presenting with virilization at puberty. Familial and cultural issues are crucial in the decision of sex of rearing at birth and on the sex re-assignment at puberty.

## ACKNOWLEDGMENT

We acknowledge Dr. Jennifer Barker for her contribution on management of the index patient.

**Ethics**

Informed Consent: It was taken.

Peer-review: External peer-reviewed.

## AUTHORSHIP CONTRIBUTIONS

Concept: Asma Deeb, Hana Al Suwaidi, Design: Asma Deeb, Fakunle Ibukunoluwa, Data Collection or Processing: Asma Deeb, Salima Attia, Hana Al Suwaidi, Analysis or Interpretation: Asma Deeb, Hana Al Suwaidi, Literature Search: Asma Deeb, Fakunle Ibukunoluwa, Writing: Asma Deeb, Salima Attia, Fakunle Ibukunoluwa.

Financial Disclosure: The authors declared that this study has received no financial support.

## Figures and Tables

**Table 1:**
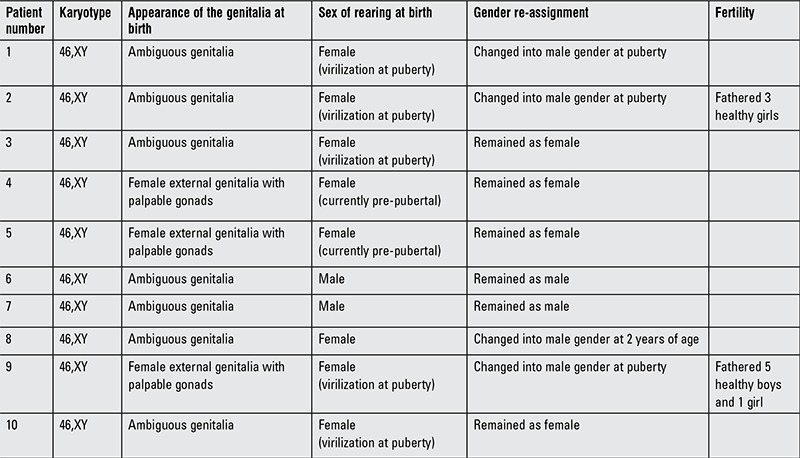
Clinical description of genital appearance at birth, sex of rearing, and gender reassignment of the 10 affected members belonging to the same family as the index patient
